# A New Real-Time PCR Test (Flora Select™) and Nugent Score for the Diagnosis of Bacterial Vaginosis During Pregnancy

**DOI:** 10.3390/microorganisms12102110

**Published:** 2024-10-21

**Authors:** Hideto Yamada, Shigeki Shimada, Hajime Ota, Yuta Kobayashi, Yoshiyuki Fukushi, Shinichiro Wada, Soromon Kataoka

**Affiliations:** 1 Center for Recurrent Pregnancy Loss, Teine Keijinkai Hospital, Sapporo 006-8555, Hokkaido, Japan; 2 Department of Obstetrics and Gynecology, Teine Keijinkai Hospital, Sapporo 006-8555, Hokkaido, Japan; y.koba1021@gmail.com (Y.K.); wa_shin_2002@yahoo.co.jp (S.W.); 3 Mommy’s Clinic Chitose, Chitose 066-0038, Hokkaido, Japan; shimashige@hotmail.co.jp; 4 Department of Obstetrics and Gynecology, Sapporo Toho Hospital, Sapporo 065-0017, Hokkaido, Japan; hjm.ohta@med.showa-u.ac.jp; 5 Department of Obstetrics and Gynecology, Hakodate Central General Hospital, Hakodate 040-8585, Hokkaido, Japan; sorokata@hakochu-hp.gr.jp

**Keywords:** bacterial vaginosis, Nugent score, preterm labor, real-time PCR, *Ureaplasma*

## Abstract

This prospective cohort study aimed to evaluate the performance of Flora select™ (FS), a newly developed real-time PCR test, for the assessment of the vaginal microbiome during early pregnancy. Five hundred and fifty-six pregnant women underwent examinations of FS, Nugent score—a Gram-staining scoring system for the diagnosis of bacterial vaginosis (BV)—and conventional bacterial culture between 8 weeks and 12 gestational weeks. Nugent scores of 0–3, 4–6, and ≥7 were found in 469 (84.2%), 41 (7.4%), and 47 (8.5%) of the women, respectively. Relative dominance rates of *Lactobacillus* species of high (≥80% medium (50%≤, <80%), and low (0.1≤, <50%), and no detection (<0.1%) were 63.0%, 8.8%, 17.1%, and 11.2%, respectively. *Gardnerella*, *Prevotella*, *Atopobium*, *Streptococcus*, *Ureaplasma*, and *Mycoplasma* species were detected in 23.9%, 17.6%, 17.1%, 7.0%, 23.0%, and 4.9% of the women, respectively. *Gardnerella* species were detected in all women with Nugent scores ≥7 and *Ureaplasma* were detected in 40.4% of them. BV-associated bacterial species were also detected in 70.7% of women with Nugent scores of 4–6. *Gardnerella*, *Prevotella*, *Atopobium*, *Streptococcus*, *Ureaplasma*, and *Mycoplasma* species were highly prevalent in women with Nugent scores ≥4 or *Lactobacillus* species <50%. FS detected *Gardnerella*, *Prevotella*, and *Atopobium* species more effectively than conventional bacterial culture. FS could determine relative dominance rates of *Lactobacillus* species in the vaginal microbiome, and simultaneously detect four kinds of BV-associated bacteria, *Ureaplasma* and *Mycoplasma* species. Therefore, FS may be clinically useful for the screening of the vaginal microbiome during pregnancy to prevent preterm birth and for the assessment of the vaginal microbiome after BV treatments.

## 1. Introduction

Preterm birth is a major cause of perinatal mortality and the long-term neurologic morbidity of newborns; the risk of preterm birth increases in pregnant women with bacterial vaginosis (BV) [1]. BV results from the stabilization or colonization of several vaginal bacteria such as *Gardnerella*, *Bacteroides*, *Ureaplasma*, and *Mycoplasma* species. To diagnose BV, classical clinical signs and symptoms of Amsel’s criteria [2] or the microscopically based Nugent score [3] have been used over the years. However, the morphologic assessment of bacteria species is time-consuming, somewhat subjective, and cannot accurately identify the pathogens [4].

The human vaginal microbiome plays a role in causing or defending against BV. *Lactobacillus* species have the beneficial function of protecting against ascending infections of microorganisms during pregnancy [5]. The vaginal microbiome profiles of healthy pregnant women shifted toward a *Lactobacillus*-dominant state during early pregnancy [6]. Decreases in the abundance of *Lactobacillus* species, and increases in BV-associated bacteria and *Ureaplasma* species in the vaginal microbiome, are associated with BV, vaginal infection, and ascending infections of microorganisms, which may cause intrauterine infections such as chorioamnionitis and preterm births [7].

Recently, molecular testing using real-time polymerase chain reaction (PCR) and 16S rRNA sequencing for BV diagnosis has been developed. A study found that log *Lactobacillus crispatus*/*Gardnerella vaginalis* was the best diagnostic indicator of BV [8]. A meta-analysis of cohort studies for the vaginal microbiome using 16S rRNA sequencing during pregnancy revealed that *Lactobacillus crispatus* reduced preterm birth, whereas *Lactobacillus iners*, *Gardnerella* and *Prevotella* species increased the risk of preterm birth [9]. A randomized clinical trial on pregnant women at low risk of preterm birth demonstrated that real-time PCR tests and treatment for BV, based on *Gardnerella* and *Atopobium* quantification, significantly reduced preterm birth rates in nulliparous women [10]. The development of culture-independent molecular diagnostics has enabled the detection of BV-associated non-cultivable bacterial species. Multiple nucleic acid amplification tests for BV diagnosis are commercially available in the United States [4]. 

This prospective cohort study evaluated the performance of Flora select™ (FS), a newly developed real-time PCR test, in the assessment of the vaginal microbiome during early pregnancy in comparison with the Nugent score and conventional bacterial culture.

## 2. Materials and Methods

### 2.1. Study Participants

This prospective cohort study was conducted between December 2023 and July 2024 according to the guidelines of the Declaration of Helsinki and was approved by the Institutional Review Board of Teine Keijinkai Hospital (No. 2-023272-00, Date of approval: 17 November 2023). Written informed consent was obtained from participants or the guardians of women under 18 years old in four hospitals/clinics. 

Pregnant women underwent examinations of FS, together with Nugent scoring—a Gram-staining scoring system for BV diagnosis—and conventional bacterial culture of vaginal swabs between 8 weeks and 12 weeks of gestation at regular maternity checkups. Two swabs were used to obtain vaginal fluid. The first swab was used for the Nugent score and microbiological culture, and the second for FS.

### 2.2. Procedures

#### 2.2.1. Nugent Score and Microbiological Culture

The Nugent scoring system evaluates bacterial morphotypes microscopically for BV diagnosis using Bartholomew and Mittwer methods (Muto Pure Chemicals Co., Ltd., Tokyo, Japan) and the Favor method (Shimadzu Diagnostics Co., Tokyo, Japan) for gram staining. Nugent scores range from 0 to 10, according to the quantitative presence of three bacterial morphotypes, which are assessed for Gram-stained vaginal fluid. The quantitative number of *Lactbacillus* morphotypes is scored 0–4, where 0 indicates the lowest amount. Small Gram-variable rods (*Gardnerella vaginalis* and *Bacteroides* morphotypes) are scored 0–4, where 4 indicates the highest amount, and curved Gram-variable rods (*Mobiluncus* morphotypes) are scored 0–2, where 2 indicates the highest amount. BV is diagnosed when Nugent scores are ≥7 [3,11]. In the present study, Nugent scores were classified into 0–3 (BV-negative), 4–6 (BV-intermediate), and ≥7 (BV-positive).

For microbiological culture, swabs of vaginal fluid were inoculated onto BD BBL™ chocolate II agar medium (Nippon Becton Dickinson Co., Ltd., Tokyo, Japan) for fastidious microorganisms—especially *Neisseria* and *Haemophilus* species; onto BD BBL™ TSAII 5% sheep blood agar medium/BTB lactose agar medium (Nippon Becton Dickinson Co., Ltd.) for *Lactobacillus* and *Streptococcus* species; onto AccuRate™ separated ABHK agar/BBE agar (Shimadzu Diagnostics Co.) for anaerobic culture and *Prevotella* species; and onto BD BBL™ CHROMagar™ Candida II agar medium (Nippon Becton Dickinson Co., Ltd.) for candida and fungus. The cultures of *Ureaplasma* and *Mycoplasma* species were not performed. Microbiological culture was performed at a temperature of 37 °C and humidity of 50–80% or 95% in CO2 incubation. 

All bacteria and fungi isolated were identified using the VITEK™ MS PRIME (bioMérieux Japan Ltd., Tokyo, Japan), which can detect almost all bacteria. 

#### 2.2.2. Real-Time PCR Test (Flora Select^TM^)

The samples for Nugent score, bacterial culture, and Flora select^TM^ (FS, Varinos Inc., Tokyo, Japan) were simultaneously obtained by swabbing the vaginal walls using two different swabs. Vaginal swab samples for FS were directly collected into a Copan eNAT collection tube (Copan Italia, Brescia, Italy). The samples were stored at room temperature until DNA extraction, which was performed within four weeks according to the manufacturer’s protocol. DNA extraction and the amplification of bacterial DNA by real-time PCR, using SYBR Green Method (TOYOBO, Osaka, Japan), was conducted by Varinos Inc. (Tokyo, Japan). The samples were treated with proteinase K of ≥600 U/mL (Kanto kagagu Co., Inc., Tokyo, Japan) and a lysozyme solution of 1.5 mg/mL (Merck KGaA, Darmstadt, Germany) for cell lysis as pretreatment. Genomic DNA was extracted using MagNA Pure 24 system (Pathogen 1000 hp 3.2 software/protocol, Roche Diagnostics GmbH, Mannheim, Germany).

The relative absolute abundance of *Lactobacillus* species was calculated by the ratio of the amount of *Lactobacillus* to the total amount of bacteria species. The cycle threshold (Ct) of each sample was compared with that of the standard curve made by diluting the genomic DNA of *Lactobacillus crispatus*. *Lactobacillus* abundance was classified into four categories, based on relative dominance rates of *Lactobacillus* species, comprising high (≥80%), medium (50%≤, <80%), low (0.1≤, <50%), and no detection (<0.1%). The detection of four BV-associated bacteria (*Gardnerella*, *Atopobium*, *Streptococcus*, and *Prevotella*) species and two miscarriage/preterm birth-associated bacteria (*Ureaplasma* and *Mycoplasma*) species was performed by multiplex PCR using specific primers for each bacterium. Primers for the four BV-associated bacteria and *Ureaplasma* were designed to amplify all known species in each genus and were targeted at the 16S or 23S rRNA gene region. Two sets of primers were designed to amplify *Mycoplasma species* including *Mycoplasma genitalium* and *hominis*, which are found in female reproductive organs.

The threshold for a positive result for each bacterium was adjusted to the threshold of a conventional bacterial culture test. The amplification reactions were performed on an CFX96 C1000 Touch Real-Time System (CFX Maestro software, Bio-Rad Laboratories, Inc., Hercules, CA, USA) using a total volume of 20 μL containing THUNDERBIRD Next SYBR qPCR Mix (TOYOBO Co., Ltd., Osaka, Japan), forward and reverse primer sets (at 0.5 μM concentration), and the extracted bacterial DNA. A no-template control and a positive control were included on each plate.

The correlation coefficient between FS and next-generation sequencing for relative dominant rates of *Lactobacillus* species was 0.995. Both positive and negative predictive values of FS for known DNA samples of the six bacteria were found to be 100%. 

### 2.3. Statistical Analysis

Categorical variables were compared using Fisher’s exact test. The prevalence of having *Lactobacillus* species <50%, BV-associated bacteria, *Ureaplasma*, and *Mycoplasma* species was compared among women with Nugent scores 0–3, 4–6, and ≥7. The prevalence of having Nugent scores ≥7/≥4, BV-associated bacteria, *Ureaplasma*, and *Mycoplasma* species was compared among women with high, medium, low, and no detection of relative dominance rates of *Lactobacillus* species. All *p*-values were two-sided, and a *p*-value < 0.05 was considered statistically significant. Statistical analyses were conducted using R software (version 4.3.2) (R Foundation for Statistical Computing, Vienna, Austria).

## 3. Results

A total of 556 pregnant women with a median age of 31 (range, 16–45) years were enrolled. Table 1 shows participant characteristics, Nugent scores, and results of FS. Of the 556 women, 19 (3.4%) had an obstetric history of only one preterm birth between 32 weeks and 36 weeks of gestation, and 119 (21,4%) had a history of miscarriage. Nugent scores of 0–3 (BV-negative), 4–6 (BV-intermediate), and ≥7 (BV-positive) were found in 468 (84.2%), 41 (7.4%), and 47 (8.5%) of the participants, respectively. Relative dominance rates of *Lactobacillus* species of high (≥80%), medium (50%≤, <80%), low (0.1≤, <50%), and no detection (<0.1%) were 63.0%, 8.8%, 17.1%, and 11.2%, respectively. BV-associated bacteria of *Gardnerella*, *Prevotella*, *Atopobium*, and *Streptococcus* species were detected in 23.9%, 17.6%, 17.1%, and 7.0% of the participants, respectively. Miscarriage/preterm birth-associated bacteria of *Ureaplasma* and *Mycoplasma* species were detected in 23.0% and 4.9% of the participants, respectively.

Table 2 shows the results of FS compared with Nugent scores. BV-associated bacteria and *Gardnerella* species were detected in all 47 women with Nugent scores ≥7, and *Ureaplasma* species was detected in 19 (40.4%) of the 47 women. BV-associated bacteria species were detected in 29 (70.7%) of 41 women with Nugent scores of 4–6. The prevalence of having *Lactobacillus* species <50%, BV-associated bacteria, *Ureaplasma*, and *Mycoplasma* species was significantly different among women with Nugent scores of 0–3, 4–6, and ≥7.

Table 3 shows the results of FS compared with the relative dominance rates of *Lactobacillus* species. BV-associated bacteria species were detected in 80 (84.2%) of 95 women with low (0.1≤, <50%) and in 41 (66.1%) of 62 women with no detection (<0.1%). The prevalence of having Nugent scores ≥7/≥4, BV-associated bacteria, *Ureaplasma*, and *Mycoplasma* species was significantly different among women with high, medium, low, and no detection of relative dominance rates of *Lactobacillus* species.

Table 4 shows the comparison between conventional bacterial culture and FS to detect BV-associated bacteria species. FS detected *Gardnerella*, *Prevotella*, and *Atopobium* species more than conventional bacterial culture. Conventional bacterial cultures detected 46 of 133 women to be FS-positive for *Gardnerella* species, only 4 of 98 women as FS-positive for *Prevotella* species, and none of 95 women as FS-positive for *Atopobium* species. However, the conventional bacterial culture detected *Streptococcus* species more than FS, although PCR primers were set for all *Streptococcus* species.

**Figure 1:**
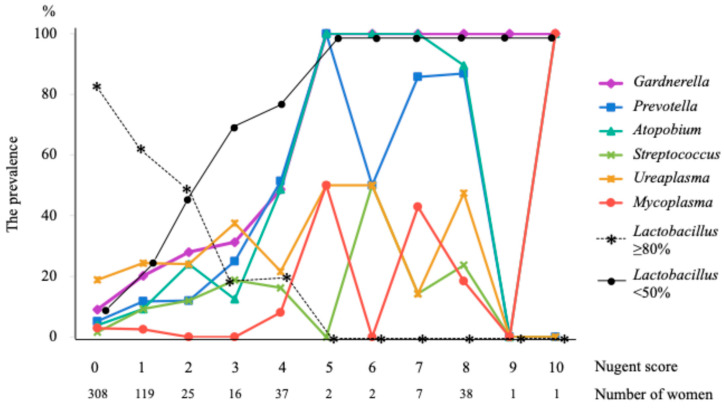
The prevalence of each bacterium according to Nugent score.

Figure 1 shows the prevalence of *Gardnerella*, *Prevotella*, *Atopobium*, *Streptococcus*, *Ureaplasma*, and *Mycoplasma* species, and relative dominance rates of *Lactobacillus* species—≥80% (high) and <50% (low and no detection)—according to the Nugent score. Of 556 women, 468 with Nugent scores of 0–3 (84.2%) had a prevalence of *Gardnerella* species of <31.3%, while 51 (9.2%) women with Nugent scores of 5–10 had a prevalence of *Gardnerella* species of 100% and a prevalence of *Atopobium* and *Prevotella* species of ≥50%, except for 2 women with Nugent scores 9–10. The prevalence of relative dominance rates of *Lactobacillus* species ≥80% (high) declined from 82.5% at Nugent score 0 to 18.8% at the score 3, and further to 0% at the scores 5–10. The prevalence of relative dominance rates of *Lactobacillus* species <50% (low and no detection) increased from 9.4% at a Nugent score of 0, to 68.8% at a score of 3, to 75.7% at a score of 4, and further to 100% at scores of 5–10. 

Of 37 women with a Nugent score of 4, *Lactobacillus* ≥80% was detected in 18.9%, *Lactobacillus* <50% in 75.7%, *Gardnerella* in 48.7%, *Atopobium* in 48.7%, and *Prevotella* in 51.4%.

## 4. Discussion

This prospective study, for the first time, evaluated the performance of Flora select™ (FS), a real-time PCR test, for the assessment of the vaginal microbiome between 8 weeks and 12 weeks of gestation in women at relatively low risk of preterm birth. This study revealed that the relative dominance rates of *Lactobacillus* species—classified as high, medium, low, and no detection—were 63.0%, 8.8%, 17.1%, and 11.2%, respectively. *Gardnerella*, *Prevotella*, *Atopobium*, *Streptococcus*, *Ureaplasma*, and *Mycoplasma* species were detected in 23.9%, 17.6%, 17.1%, 7.0%, 23.0%, and 4.9% of the pregnant women, respectively (Table 1). *Gardnerella* species were detected in all women with a Nugent score ≥7, and 40.4% of them had *Ureaplasma* species. BV-associated bacteria species were also detected in 70.7% of women with Nugent scores of 4–6. The prevalence of *Gardnerella*, *Prevotella*, *Atopobium*, *Streptococcus*, *Ureaplasma*, and *Mycoplasma* species was very high in women with BV scores ≥4 (Table 2) or *Lactobacillus* species <50% (Table 3). FS also detected *Gardnerella*, *Prevotella*, and *Atopobium* species more effectively than conventional bacterial culture (Table 4). FS could determine relative dominance rates of *Lactobacillus* species in the vaginal microbiome, and simultaneously detect four kinds of BV-associated bacteria, particularly *Ureaplasma* and *Mycoplasma* species, which cannot be detected by Gram staining or conventional bacterial culture. Therefore, FS may be clinically useful for the screening of the vaginal microbiome during pregnancy to prevent preterm labor and for the assessment of the vaginal microbiome after BV treatments.

Although the routine screening or treatment of BV for asymptomatic women at low risk for preterm birth is generally not recommended [12], there is evidence from one trial that BV screening and treatment programs for pregnant women before 20 weeks of gestation reduces preterm birth and preterm low birthweight [13,14]. In the present study, none of the women with Nugent scores 5–10 had *Lactobacillus* ≥80% (high), but all had *Lactobacillus* <50% and *Gardnerella* species. (Figure 1). Therefore, all women with Nugent scores 5–10 should receive treatment for BV during pregnancy, while women with a Nugent score of 4 and with positive tests for BV-associated bacteria and *Lactobacillus* <50% can be treated for BV. 

In the 556 women, 73 (13.1%)/80 (14.4%) had *Gardnerella*/BV-associated bacteria species with low levels of *Lactobacillus* (0.1≤, <50%) and 34 (6.1%)/41 (7.4%) had *Gardnerella*/BV-associated bacteria species with no detection (<0.1%) of *Lactobacillus* species (Table 3). When FS is used for the screening of the vaginal microbiome during the first trimester, these women are prime candidates for antibiotic therapy to prevent preterm labor.

*Ureaplasma* species are the most frequently isolated microorganisms from the amniotic fluid and placentae of women who deliver preterm, and are also associated with miscarriages, neonatal respiratory diseases, and chorioamnionitis [15]. A retrospective cohort study found that the presence of *Ureaplasma* species in the vaginal microbiome at <11 weeks of gestation was causally associated with late miscarriages and early preterm birth [16]. Recently, a prospective cohort study of pregnant women with threatened miscarriage/preterm labor demonstrated that the presence of *Ureaplasma* species and decreased relative dominance rate of *Lactobacillus* species in the vaginal microbiome predicted a high possibility of subsequent preterm birth [17]. In the uterine endometrium microbiome of nonpregnant women with recurrent pregnancy loss, the presence of *Ureaplasma* species increased the risk of the miscarriage of fetuses with normal chromosomes in their subsequent pregnancies, and the presence of *Ureaplasma* and *Gardnerella* species and a decreased relative dominance rate of *Lactobacillus* species increased the risk of preterm birth in the subsequent pregnancies [18]. These prospective cohort studies have revealed that the presence of *Ureaplasma* species in the vagina during pregnancy and in the uterine endometrium before pregnancy increased the risk of adverse pregnancy outcomes. Although *Ureaplasma* species cannot be detected by Gram staining or conventional bacterial culture, the present study using FS identified *Ureaplasma* species in the vaginal microbiome in 21.2%, 24.4%, and 40.4% of pregnant women with Nugent scores 0–3, 4–6, and ≥7, respectively (Table 2). Because metronidazole, as a standard treatment for BV, is not effective against *Ureaplasma* species, these women could be treated further with macrolides. FS may be clinically useful because it can detect *Ureaplasma* species and BV-associated bacteria simultaneously.

This prospective study has several limitations. All participants were Japanese; the human vaginal microbiome changes according to race, diet, living environment, and life cycle stage. Differences in *Lactobacillus* species (*L. crispatus*, *gasseri*, *jensenii*, and *iners*) were not assessed. Repeated FS tests were needed to confirm the results. Pregnancy outcomes (miscarriage, preterm birth, and term delivery) of participants in relation to results of FS and Nugent score were not evaluated. The efficacy of prophylactic antibiotic therapy or the cost-effectiveness of FS as a screening compared to Nugent score were not determined. These should be clarified in further investigations, and the cohort study is now in progress.

## 5. Conclusions

This study demonstrated that FS could determine the relative dominance rates of *Lactobacillus* species in the vaginal microbiome, and simultaneously detect four kinds of BV-associated bacteria, together with *Ureaplasma* and *Mycoplasma* species. FS may be clinically useful for the screening of the vaginal microbiome during pregnancy to prevent preterm birth and for the assessment of the vaginal microbiome after BV treatments. When FS is used for the screening of the vaginal microbiome during the first trimester, women having *Gardnerella*/BV-associated bacteria species with low/no detection of *Lactobacillus* species are prime candidates for treatment with antibiotic therapy to prevent preterm birth.

## Figures and Tables

**Table 1:** Participant characteristics, Nugent score, and results of Flora select™.

Characteristics of Participants *n* = 556	Median	Range
Age, years	31	16–45
Body mass index, kg/m^2^	21.3	15.4–37.8
Gravidity	2	1–8
Parity	1	0–6
Number of previous miscarriages	0	0–4
Number of previous preterm births	0	0–1
Gestational week (w) and day (d) of examinations	10w4d	8w0d–12w6d
	**Number**	**%**
Number of women with a history of miscarriage	119	21.4
Number of women with a history of preterm birth	19	3.4
**Nugent score**	**Number**	**%**
0–3	468	84.2
4–6	41	7.4
≥7	47	8.5
**Flora select™**	**Number**	**%**
Relative dominance rate of *Lactobacillus* species		
High (≥80%)	350	63.0
Medium (50%≤, <80%)	49	8.8
Low (0.1≤, <50%)	95	17.1
No detection (<0.1%)	62	11.2
Presence of BV-associated bacteria species		
*Gardnerella*	133	23.9
*Prevotella*	98	17.6
*Atopobium*	95	17.1
*Streptococcus*	39	7.0
Miscarriage/preterm birth-associated bacteria species		
*Ureaplasma*	128	23.0
*Mycoplasma*	27	4.9

BV, bacterial vaginosis.

**Table 2:** Results of Flora select™ compared with Nugent scoring.

Nugent Score	Number	*Lactobacillus* Species <50% (%)	Presence of BV-Associated Bacteria Species (%)	Presence of ≥Two BV-Associated Bacteria Species (%)	BV-Associated Bacteria Species				Miscarriage/Preterm Birth-Associated Bacteria Species	
					*Gardnerella* (%)	*Prevotella* (%)	*Atopobium* (%)	*Streptococcus* (%)	*Ureaplasma* (%)	*Mycoplasma* (%)
0–3	468	16.9	16.7	10.7	13.7	7.9	6.6	4.7	21.2	2.6
4–6	41	78.0	70.7	61.0	53.7	53.7	53.7	17.1	24.4	9.8
≥7	47	97.9	100.0	95.7	100.0	83.0	89.4	21.3	40.4	23.4
Fisher’s exact test		*p* < 0.01	*p* < 0.01	*p* < 0.01	*p* < 0.01	*p* < 0.01	*p* < 0.01	*p* < 0.01	*p* = 0.014	*p* < 0.01

BV, bacterial vaginosis.

**Table 3:** Nugent score and results of Flora select™ compared with relative dominance rates of *Lactobacillus* species.

Relative Dominance Rate of *Lactobacillus* Species	Number	Nugent Scores ≥7/Nugent Scores ≥4 (%)	Presence of BV-Associated Bacteria Species (%)	Presence of ≥Two BV-Associated Bacteria Species (%)	BV-Associated Bacteria Species				Miscarriage/Preterm Birth-Associated Bacteria Species	
					*Gardnerella* (%)	*Prevotella* (%)	*Atopobium* (%)	*Streptococcus* (%)	*Ureaplasma (%)*	*Mycoplasma (%)*
High (≥80%)	350	0.0/2.0	3.1	2.0	2.3	1.7	1.4	0.9	18.3	1.4
Medium (50%≤, <80%)	49	2.0/6.1	44.9	22.5	36.7	20.4	18.4	8.2	28.6	10.2
Low (0.1≤, <50%)	95	30.5/45.3	84.2	72.6	76.8	57.9	63.2	17.9	33.7	13.7
No detection (<0.1%)	62	27.4/56.5	66.1	53.2	54.8	43.6	33.9	24.2	29.0	6.5
Fisher’s exact test		*p* < 0.01/*p* < 0.01	*p* < 0.01	*p* < 0.01	*p* < 0.01	*p* < 0.01	*p* < 0.01	*p* < 0.01	*p* < 0.01	*p* < 0.01

BV, bacterial vaginosis.

**Table 4:** BV-associated bacteria species detected by conventional bacterial culture and Flora select™.

*Gardnerella* Species	Bacterial Culture		Total	*Prevotella* Species	Bacterial Culture		Total
	Positive	Negative			Positive	Negative	
**Flora select™**				**Flora select™**			
**Positive**	46	87	133	**Positive**	4	94	98
**Negative**	5	418	423	**Negative**	0	458	458
**Total**	51	505	556	**Total**	4	552	556
***Atopobium* Species**	**Bacterial Culture**		**Total**	***Streptococcus* Species**	**Bacterial Culture**		**Total**
	**Positive**	**Negative**			**Positive**	**Negative**	
**Flora select™**				**Flora select™**			
**Positive**	0	95	95	**Positive**	16	23	39
**Negative**	0	461	461	**Negative**	55	462	517
**Total**	0	556	556	**Total**	71	485	556

BV, bacterial vaginosis.

## Data Availability

The data underlying this study cannot be shared publicly for privacy reasons. The datasets generated and/or analyzed during the current study are available from the corresponding author on reasonable request.
